# Dosimetric impact of target volume anatomical parameters on radiotherapy after right-sided breast-conserving surgery

**DOI:** 10.3389/fonc.2026.1944304

**Published:** 2026-10-26

**Authors:** Lu Zou, Zeping Deng, Xingliang Liao, Ling Duan, Xuefen Liu

**Affiliations:** 1 Department of Oncology, The People’s Hospital of Rongchang District, Chongqing, China; 2 Department of Pediatrics, Chongqing University Three Gorges Hospital, Chongqing, China

**Keywords:** anatomical parameters, dosimetry, organs at risk, radiotherapy, right-sided breast-conserving surgery for breast cancer

## Abstract

**Objective:**

To investigate the dosimetric effects of target volume Anatomical Parameters on organ at risk (OAR) following breast-conserving surgery and subsequent radiotherapy in patients with right-sided breast cancer.

**Methods:**

A retrospective study was conducted on 33 patients who underwent post-breast-conserving surgery radiotherapy. The transverse diameter (TD), oblique diameter (OD), and the ratio of transverse diameter to oblique diameter (TOR) were measured at the nipple-center plane for all patients. Bivariate Pearson correlation analysis was used to examine the effects of TD, OD, and TOR on V_20Gy_, V_5Gy_, D_mean_ of the affected lung; D_mean_ and V_5Gy_ of heart; D_mean_ of the right coronary artery (RCA); D_mean_ of the left anterior descending artery (LAD); V_5Gy_ of the contralateral lung; and D_mean_ of the contralateral breast.

**Results:**

OD and TD showed significant correlations with V_20Gy_, V_5Gy_, and D_mean_ of the affected lung (*0.5 < R < 0.7, P < 0.01*); The TD was significantly correlated with Heart_D_mean_ (*R = 0.58, P < 0.01*), strongly correlated with RCA_D_mean_ (*R = 0.45, P < 0.01*), and strongly correlated with LAD_D_mean_ (*R = 0.42, P < 0.05*); The TOR was significantly correlated with Heart_D_mean_ (*R = 0.47, P < 0.01*), strongly correlated with LAD_D_mean_ (*R = 0.47, P < 0.01*).

**Conclusion:**

Exploratory observational thresholds from our cohort: target volume with TD >8.00 cm or OD >19.56 cm indicates higher lung dose risk; TD >8.00 cm or TOR >0.47 indicates higher cardiac dose risk. These thresholds were not optimized by ROC analysis and cannot be directly used for clinical risk stratification; prospective validation is needed. Clinical planning should minimize heart and lung radiation.

## 1 Introduction

Breast cancer is one of the most common malignant tumors among women, accounting for approximately 30% of new cancer cases in women (1), Its mortality rate among women is second only to that of lung cancer (2). In China, with changes in lifestyle, increased screening coverage, and an aging population, both the incidence and mortality rates of breast cancer are on a continuous upward trend. Treatment strategies for early-stage breast cancer have shifted from traditional radical surgery to a model combining breast-conserving surgery with postoperative radiotherapy. Extensive evidence-based medical data indicates that postoperative radiotherapy following breast-conserving surgery achieves outcomes comparable to or even superior to those of radical surgery in terms of local control rates, distant metastasis rates, and overall survival (3), it has now become the standard treatment regimen for early-stage breast cancer. However, while radiotherapy achieving local tumor control, it inevitably causes radiation damage to adjacent normal tissues. With the significant prolongation of patient survival, late-onset injury associated with radiation therapy has become increasingly prominent and is now a key factor limiting long-term quality of life in patients (4). Vital organs adjacent to the breast radiation treatment field such as the lungs, heart, and contralateral breast are susceptible to radiation-induced damage. Among these, radiation-induced lung injury(RILI) and radiation-induced heart disease (RIHD) is the most common complication of breast cancer radiation therapy (5). The adoption of volumetric arc intensity-modulated radiation therapy (VMAT) has not only accelerated the use of deep inspiration breath hold (DIBH) techniques in breast cancer radiotherapy but has also significantly reduced cardiac dose, effectively lowering the incidence of radiation-induced cardiac injury (6), some studies also suggest that hypofractionated radiotherapy can reduce cardiac dose (7). The dose to organs at risk (OARs) remains closely related to various factors, including patient anatomical characteristics and respiratory motion patterns (8). A thorough investigation of the influence of key target volume anatomical parameters on OAR dose delivery holds significant clinical value for optimizing radiation treatment plans and achieving precise dose constraints. Currently, research on the correlation between anatomical parameters of the target volume such as transverse diameter (TD), oblique diameter (OD), and the ratio of transverse diameter to oblique diameter (TOR) and dosimetric indicators of OAR is relatively scarce. Existing literature has not systematically elucidated the predictive value of these morphological parameters for cardiac and pulmonary dose. Therefore, this study aims to conduct a retrospective analysis of the morphological characteristics of the treatment field in patients undergoing postoperative radiotherapy for right-sided breast cancer. We will investigate the correlation between the treatment field’s TD, OD, and TOR and key OAR dosimetric indicators. The objective is to explore quantitative associations between treatment-field geometric characteristics and OAR doses, providing individualized dose-limiting references for clinical planning design, thereby further reducing the incidence of radiation-induced damage reactions and improving patients’ long-term prognosis.

## 2 Materials and methods

### 2.1 General clinical data

This study retrospectively enrolled female patients who received postoperative radiotherapy after right breast-conserving surgery from January 2025 to August 2025. The detailed inclusion and exclusion criteria were formulated as follows. Inclusion criteria (1): Female patients aged 18–70 years (2); Postoperative pathological diagnosis was breast cancer (3); Patients with ductal carcinoma *in situ* underwent breast-conserving surgery with or without sentinel lymph node biopsy, while patients with invasive breast cancer received breast-conserving surgery combined with sentinel lymph node biopsy or axillary lymph node dissection (4); Postoperative tumor stage was T_1-2_N_0-1_M_0_ (5); No neoadjuvant therapy administered, and standardized postoperative systemic therapy completed (6); No contraindications to thoracic radiotherapy. Exclusion criteria (1): A history of previous radiotherapy to the chest wall or adjacent thoracic regions (2); Complicated with other malignant tumors or severe non-neoplastic systemic diseases (3); Positive surgical margins or patients with ≥3 positive sentinel lymph nodes who did not receive subsequent axillary dissection (4); Receipt of neoadjuvant therapy before surgery (5); Pregnancy or lactation status (6); Patients unable to complete the whole course of radiotherapy.

A total of 33 eligible female patients were included in the final analysis. The patients’ age ranged from 31 to 65 years, with a median age of 47 years. In terms of tumor staging, 22 patients were classified as T_1_ stage and 11 as T_2_ stage; 31 patients were N_0_ stage and 2 were N_1_ stage. All patients received a uniform radiotherapy regimen with a prescription dose of 43.5 Gy in 15 fractions for the planning target volume (PTV) of whole breast and a simultaneous integrated boost dose of 49.5 Gy in 15 fractions for the boosted PTV (PTV_boost_). Planning was performed using the Elekta Monaco 6.0 planning system. VMAT was employed for all patients. Specific planning parameters were as follows: field angles ranging from 200° to 60°, with a maximum of two arcs per beam; Auto Flash set to 2 cm; a calculation grid of 3 mm; and a calculation uncertainty of 1% per iteration. All other parameters were identical. All plans were normalized such that the prescribed dose covered 95% of the PTV volume.

### 2.2 Localization scanning and evaluation indicators

All geometric measurements were performed on the nipple-central slice of simulation CT using built-in tools within the Monaco treatment planning system, where the TD and OD were measured and documented. All measurements were completed by one experienced radiation oncology physicist. Ten randomly selected cases were re-measured after a two-week interval by the same operator to assess intra-observer reproducibility, as shown in **Figure 1**. The following critical organ assessment metrics were calculated for plans meeting clinical requirements: V_20Gy_, V_5Gy_, and D_mean_ for the affected lung; D_mean_, V_5Gy_ For heart; D_mean_ for the right coronary artery (RCA) and the left anterior descending artery (LAD);V_5Gy_ of the contralateral lung; and V_5Gy_ for the contralateral breast.

**Figure 1:**
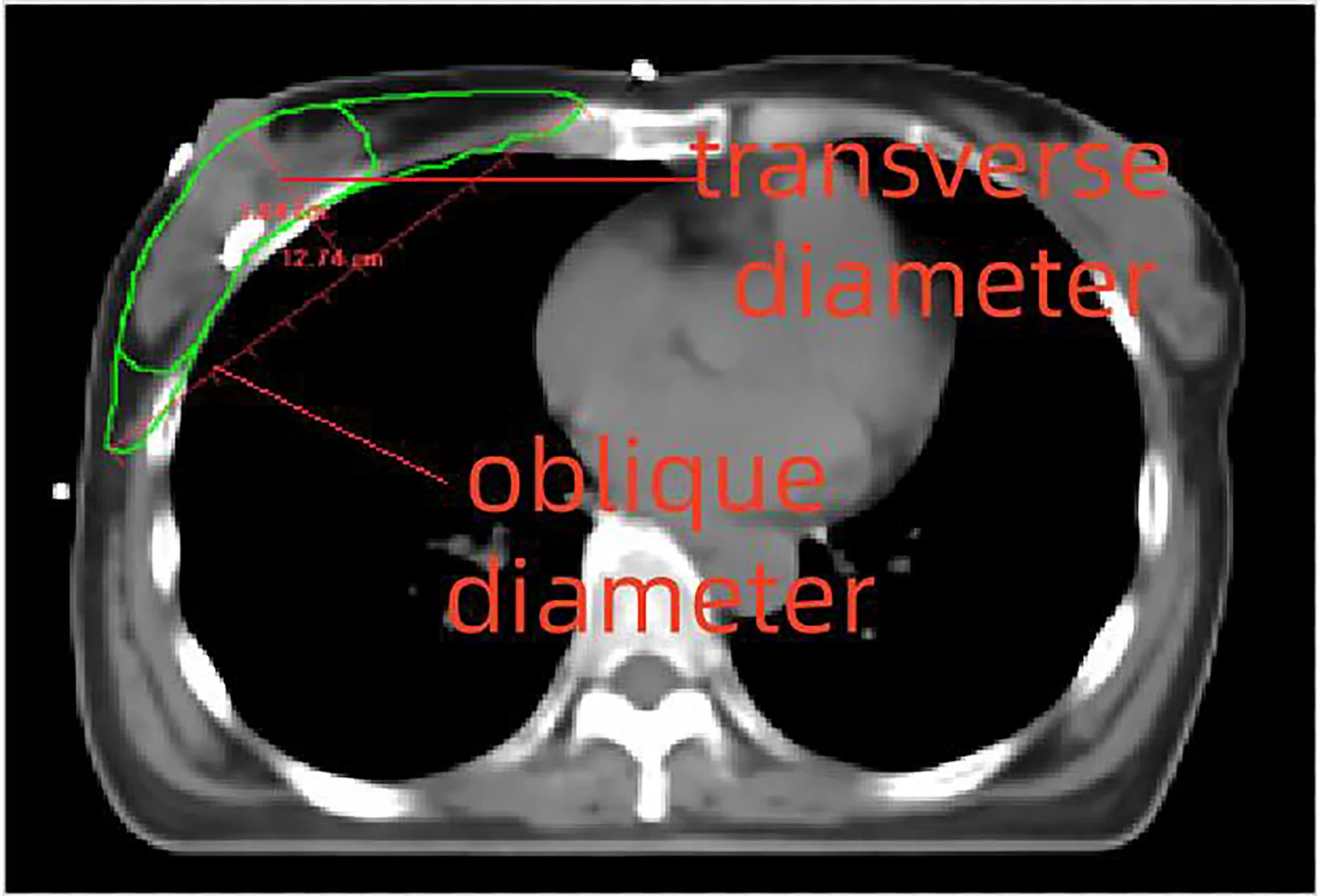
Schematic diagram of the TD and OD.

### 2.3 Statistical analyses

Statistical analysis was performed using SPSS 22.0. Quantitative data are expressed as mean *±* standard deviation (* $\bar{\text{x}}$ ± s*). The correlation between the TD, OD, and TOR of the target area and the evaluation indices was assessed using the bivariate Pearson correlation test. To mitigate multiple-comparison bias arising from 66 pairwise correlation tests, the Benjamini-Hochberg false discovery rate (FDR) correction was applied. FDR-adjusted q-values were reported, and statistical significance was defined as q-value < 0.05. Correlation heatmaps were generated using Origin 2024, and linear regression was performed for the evaluation indices and the feature parameters showing FDR-corrected significant correlations. A p-value of <0.05 was considered statistically significant.

## 3 Results

### 3.1 Target volume anatomical parameters and OAR dosimetric indices

The statistical results for target volume anatomical parameters and dosimetric parameters of OAR in 33 patients with right-sided breast cancer are shown in **Table 1**.

**Table 1:** Target volume anatomical parameters and dosimetric indices of OAR.

Target volume anatomical parameters and dosimetric indices of OAR	$\bar{x}$ ± s
Target volume parameters	
TD(cm)	4.96 ± 0.99
OD(cm)	15.92 ± 1.69
TOR	0.31 ± 0.05
Dosimetric indices of OAR	
V_20Gy_ of the affected lung(%)	14.77 ± 3.48
V_5Gy_ of the affected lung(%)	31.39 ± 4.99
D_mean_ of the affected lung(Gy)	8.12 ± 1.46
D_mean_ of heart(Gy)	1.46 ± 0.39
V_5Gy_ of heart(%)	0.79 ± 1.64
RCA_D_mean_(Gy)	2.67 ± 0.65
LAD_D_mean_(Gy)	1.55 ± 0.67
D_mean_ of the contralateral breast (Gy)	1.68 ± 0.65
V_5Gy_ of the contralateral breast(%)	0.09 ± 0.22

### 3.2 The analysis results of the correlation between target volume anatomical parameters and dosimetric parameters of organs at risk

Both OD and TD showed significant positive correlations with V_20Gy_, V_5Gy_, and D_mean_ in the affected lung (*0.5 < R < 0.7, P < 0.01*); The TD showed a significant positive correlation with Heart_D_mean_ (*R = 0.58, P < 0.01*), a strong positive correlation with RCA_D_mean_ (*R = 0.45, P < 0.01*), and a strong positive correlation with LAD_D_mean_ (*R = 0.42, P < 0.05*); The TOR showed a significant positive correlation with cardiac Dmean (R = 0.47, q< 0.01), a strong positive correlation with left coronary Dmean (R = 0.47, q< 0.01), and a moderately strong positive correlation with right coronary Dmean (R = 0.35, P = 0.046, q = 0.087, non-significant after FDR correction).The heatmap illustrating the correlations between dosimetric parameters and feature parameters is shown in **Figure 2**.

**Figure 2:**
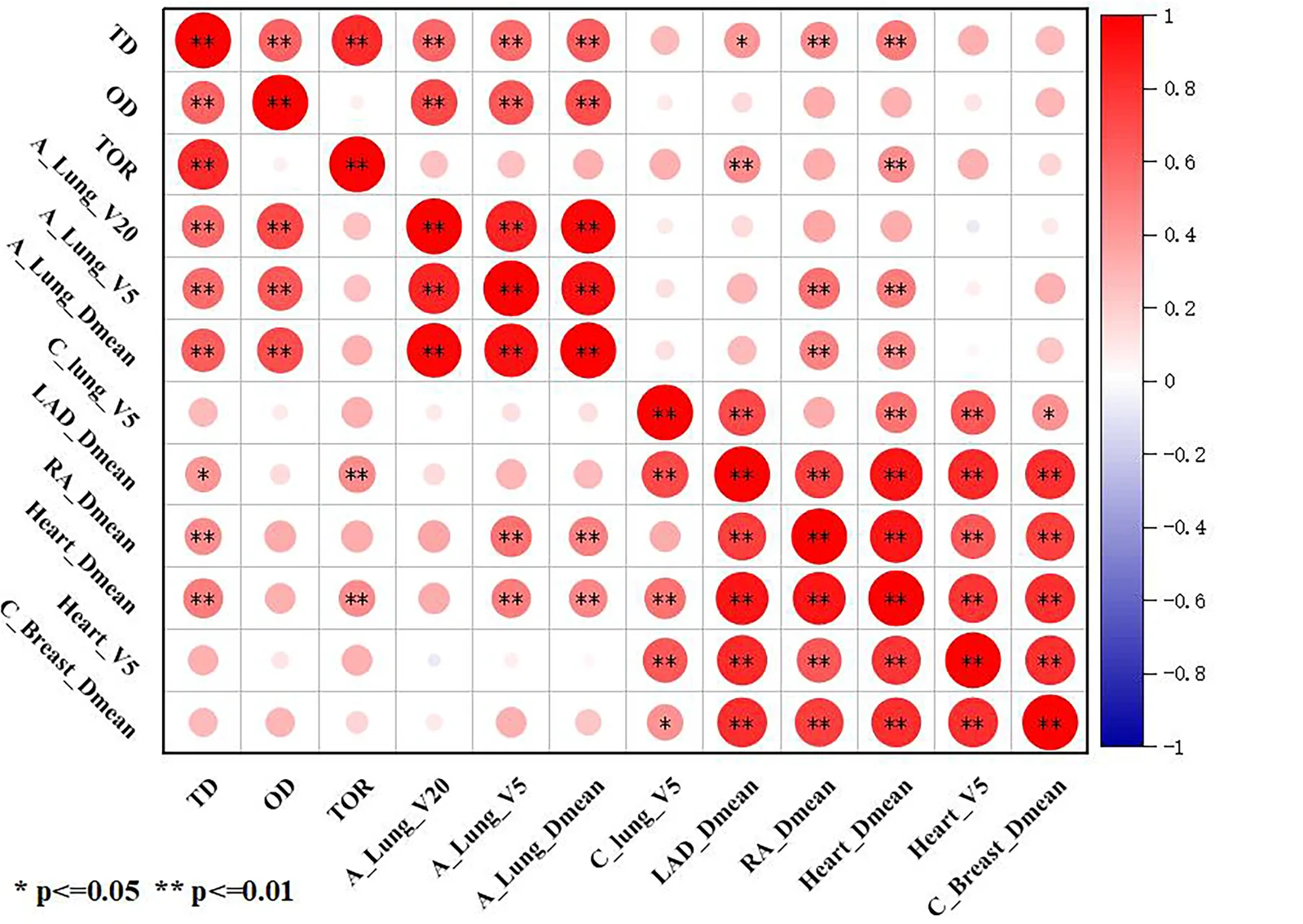
Heatmap of the correlation between dosimetric Indices and anatomical parameters. TD, Transverse diameter; OD, Oblique diameter; TOR, the ratio of transverse diameter to oblique diameter; A_Lung_V_20Gy_, V_20Gy_ of the affected lung; A_Lung_V_5Gy_, V_5Gy_ of the affected lung; A_Lung_D_mean_, D_mean_ of the affected lung; C_Lung_V_5Gy_, V_5Gy_ of the contralateral lung; LAD_D_mean_, D_mean_ of the left anterior descending artery; RCA_D_mean_, D_mean_of the right coronary artery; Heart_D_mean_, D_mean_ of heart; Heart_V_5Gy_, V_5Gy_ of heart; C_Breast_Dmean, D_mean_ of the contralateral breast.

### 3.3 The results of linear fitting

A linear regression analysis was performed on two correlated parameters. As the OD and TD increased, the V20Gy, V5Gy, and Dmean values for the affected lung also increased. In particular, when the OD exceeded 19.56 cm, the V20Gy, V5Gy, and Dmean values for the affected lung exceeded 20%, 40%, and 10 Gy, respectively; when the TD exceeded 8.00 cm, the V20Gy, V5Gy, and Dmean in the affected lung exceed 20%, 40%, and 10 Gy, respectively; the Dmean in the heart exceeds 2 Gy, LAD_Dmean exceeds 2.4 Gy, and RCA_Dmean exceeds 3.5 Gy. When the TOR is >0.47, the Dmean in the heart exceeds 2 Gy. We emphasize that these cut-offs should be regarded as exploratory reference values and require further external-cohort validation before routine clinical application. The linear fit is shown in **Figure 3**.

**Figure 3:**
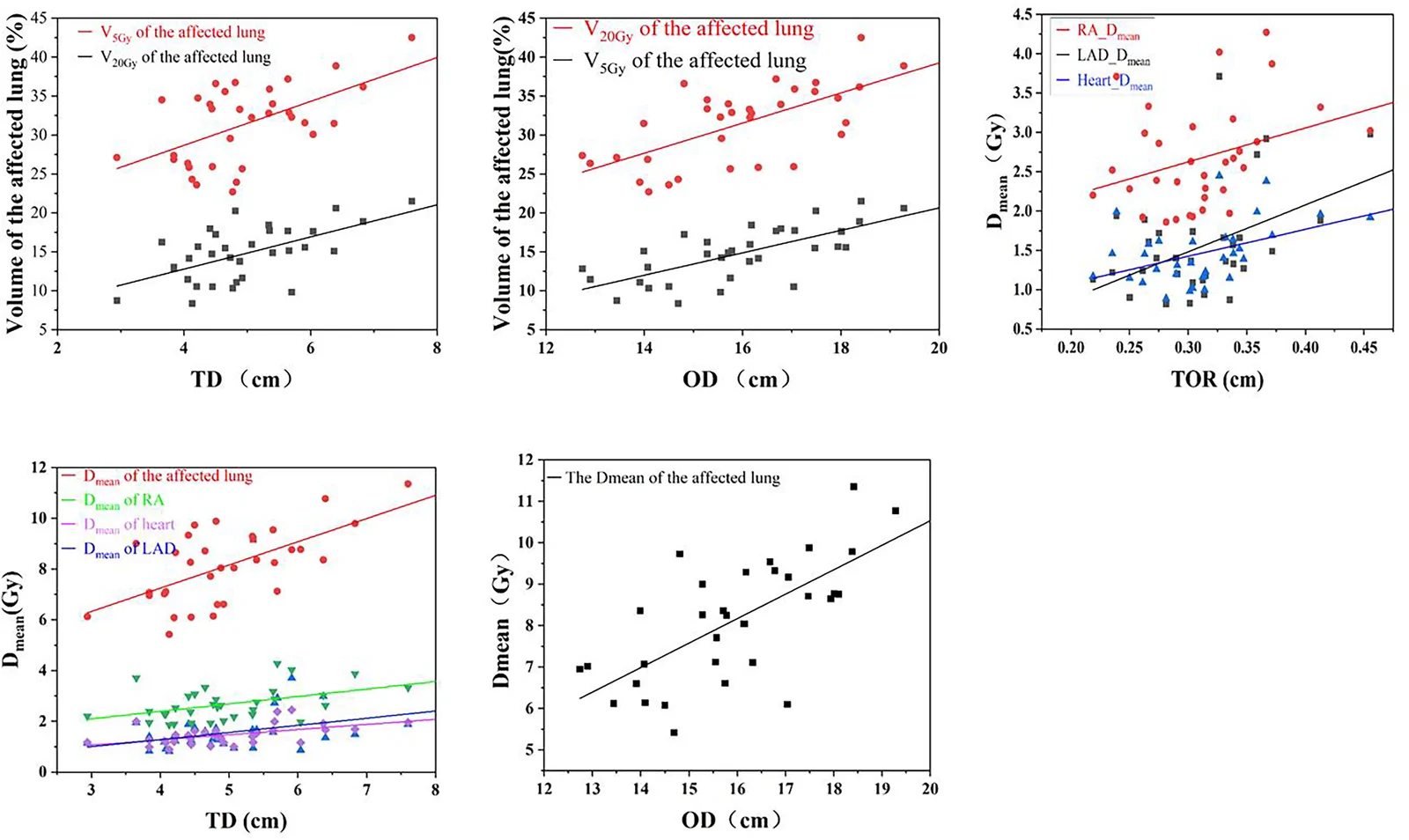
Linear fit graph of correlation metrics.

## 4 Discussion

With the continuous advancement of intensity-modulated radiation therapy techniques, VMAT can effectively improve the dose volume to the target and enhance dose homogeneity in the target area (9, 10). Post-breast-conserving surgery (BCS) radiation therapy can significantly reduce the local recurrence rate. Whole-breast radiation therapy following BCS, as the standard treatment for reducing local recurrence, has been widely proven to be effective. However, radiation-induced adverse effects, such as cardiopulmonary damage and the long-term cancer risk in the contralateral breast, remain the primary barriers to treatment benefits. Dosimetric parameters for OAR have become core indicators for evaluating the quality of radiation therapy plans. The incidence of radiation pneumonitis is closely related to the volume of lung tissue exposed to radiation, with V_20Gy_, V_5Gy_, and D_mean_ being clinically recognized predictive indicators (11–13). Regarding cardiac injury, D_mean_ and V_5Gy_ are important parameters for assessing RIHD^14^, while recent studies have further confirmed that the mean dose to the LAD and RCA is significantly associated with the incidence of coronary artery disease (14, 15). In the optimization of radiotherapy plans for breast cancer, cardiac and pulmonary doses are influenced by multiple factors, including patient anatomical characteristics, field angles, and optimization algorithms. This study innovatively focuses on the correlation between target volume geometric parameters and cardiac and pulmonary dosimetry, aiming to provide a theoretical basis for individualized dose prediction from an anatomical and geometric perspective.

This study found that TD and OD of the target volume were significantly positively correlated with V_20Gy_, V_5Gy_, and D_mean_ in the affected lung, indicating that as the TD and OD of the target volume increased, the irradiated volume of the affected lung correspondingly increased. This result is consistent with the fundamental principles of radiation physics: an increase in the geometric dimensions of the target volume directly leads to an increase in the volume of lung tissue traversed by the radiation beam, a phenomenon particularly evident in the low-dose region (V_5Gy_), This finding is largely consistent with the conclusion of a study by Mou Yanhong et al. (16) that the length of the esophageal cancer target volume was positively correlated with lung V_20Gy_. The cardiac dosimetric parameters in this study were far below the NCCN (17) guidelines’ dose limits for the heart and cardiac substructures in right-sided breast cancer, suggesting that strict limitations may have been overlooked during clinical treatment planning. However, some studies suggest that for every 1 Gy increase in the mean cardiac dose, the risk of long-term cardiac damage increases by 7.4% (18, 19), and other studies have confirmed a correlation between cardiac D_mean_ and the incidence of myocardial infarction (20). In contrast, the effect of the TD on cardiac dosimetric parameters in this study was significant, showing a positive correlation with Heart_ D_mean_, RCA_D_mean_, and LAD_Dmean, suggesting that the transverse extent of the target volume is a key factor determining the dose of heart.

The TOR parameter, proposed for the first time in this study, has significant value for dosimetric prediction. The results show that the TOR is significantly positively correlated with D_mean_ for the heart, LAD_D_mean_, and RCA_D_mean_. Although the correlation coefficients are slightly lower than those of individual geometric parameters, this indicates to some extent that this ratio can effectively reflect the comprehensive influence of the target region’s spatial morphology on the dose of heart. From an anatomical-geometric perspective, an increase in the TOR implies a corresponding increase in the curvature of the target region, leading to greater overlap between the beam incidence angle and the anatomical positions of the heart and the affected lung. Theoretically, the TOR should be correlated with cardiopulmonary dosimetry. However, no statistical correlation was observed between the TOR and the affected lung dose parameters. This negative result may stem from the following reasons: ① as a relatively “superficial” OAR, the irradiated volume of lung tissue is primarily influenced by the overall size of the target area and is less sensitive to changes in the spatial morphology of the target area; ② the sample size in this study was limited, failing to detect a weak correlation that may actually exist. Future studies should expand the sample size and utilize multicenter data for further validation.

The D_mean_ of the healthy breast showed no correlation with the geometric parameters of any target region, a result consistent with expectations. Since the healthy breast is located in the contralateral thoracic cavity and is relatively distant from the affected target region, the radiation dose it receives during conventional tangential fields and VMAT techniques primarily stems from scattered radiation and beam leakage, and is not directly related to the geometric dimensions or shape of the target region. This suggests that protection of the contralateral breast should focus on optimizing gantry angles to avoid the breast and incorporating dose-shading structures.

The clinical significance of this study is that the quantitative relationships between breast target-volume geometric parameters and cardiopulmonary doses offer a simple, practical assessment tool prior to post-breast-conserving-surgery radiotherapy. Clinically, patients with oblique diameter >19.56 cm or transverse diameter >8.00 cm are at risk of elevated ipsilateral lung dose. Patients with transverse diameter >8.00 cm or TOR >0.47 may have increased doses to the heart and cardiac sub-structures. For these patients, DIBH combined with optical-guidance technique can be used to enlarge lung volume and shift the heart away from the target, thereby lowering cardiac dose (21). Every effort should be made to minimize cardiopulmonary irradiation while guaranteeing sufficient target coverage. Moreover, the quantitative associations from the present work may be incorporated into the automatic optimization pipeline of radiotherapy planning systems for early OAR-dose warning and intelligent plan-quality evaluation.

In conclusion, the clinical significance of this study lies in the fact that by establishing a quantitative relationship between the geometric parameters of the breast target volume and cardiopulmonary doses, it provides a simple and practical assessment tool for dose prediction prior to post-breast-conserving surgery radiotherapy. In clinical practice, for patients with a diagonal diameter >19.56 cm or a transverse diameter >8.00 cm, the risk of pulmonary dose escalation can be predicted. For patients with a transverse diameter >8.00 cm or a TOR >0.47, an increased risk of cardiac dose can be predicted. During clinical treatment planning, cardiac and pulmonary doses should be minimized as much as possible while ensuring adequate coverage of the target volume. Furthermore, the quantitative associations observed in the present work may provide preliminary references for OAR dose warning; formal prediction models require further development and external-cohort validation before integration into radiotherapy planning systems.

This study has several limitations. First, it is a single-center retrospective investigation with a relatively small sample size (n = 33). Only correlative and quantitative association analyses were performed, and no fully validated clinical prediction model was established. Although the Benjamini-Hochberg FDR correction was applied to account for multiple-comparison bias, these correlative findings should be interpreted with caution. Second, all exploratory cut-off values were derived exclusively from the present single-center dataset without validation in independent external cohorts. Third, inter-observer variability for anatomical measurements was not assessed, which might introduce potential measurement bias. Notably, the thresholds (TD > 8 cm, OD > 19.56 cm, TOR > 0.47) were generated from linear observations of correlation analyses rather than optimized via ROC curve analysis. Therefore, these cut-offs cannot be directly implemented for clinical patient risk stratification. Larger-sample multicenter studies as well as prospective cohort validation and stratified analyses are warranted to verify our findings, refine these reference thresholds, and develop robust prediction tools suitable for clinical practice.

## Data Availability

The original contributions presented in the study are included in the article/supplementary material. Further inquiries can be directed to the corresponding authors.

## References

[1] Sun D Li H Cao M He S Lei L Peng J . Cancer burden in China: trends, risk factors and prevention. Cancer Biol Med. (2020) 17:879–95. doi: 10.20892/j.issn.2095-3941.2020.0387 33299641 PMC7721090

[2] Siegel RL Miller KD Wagle NS Jemal A . Cancer statistics, 2023. Ca: A Cancer J For Clin. (2023) 73:17–48. doi: 10.3322/caac.21763 36633525

[3] Lei S Zheng R Zhang S Wang S Chen R Sun K . Global patterns of breast cancer incidence and mortality: A population-based cancer registry data analysis from 2000 to 2020. Cancer Commun (Lond). (2021) 41:1183–94. doi: 10.1002/cac2.12207 34399040 PMC8626596

[4] Lin Y Sun J Qin Q Wu H Huang X L C . Application of protective effect of postoperative radiotherapy on organs at risk of breast cancer. China Med Devices. (2024) 39:147–52,63. doi: 10.3969/j.issn.1674-1633.2024.07.025

[5] Hanania AN Mainwaring W Ghebre YT Hanania NA Ludwig M . Radiation-induced lung injury: assessment and management. Chest. (2019) 156:150–62. doi: 10.1016/j.chest.2019.03.033 PMC8097634 30998908

[6] Wang Y Tang B Zhang D Liu C . Clinical application of optical surface imaging system in deep inspiration breath hold radiotherapy for left-sided breast cancer. J Cancer Control Treat. (2025) 38:648–54. doi: 10.3969/j.issn.1674-0904.2025.07.013

[7] Huang M Tang C Wang X Chi J Xie R . Study on the safety of large-segment radiotherapy after radical surgery for left breast cancer and the evaluation of heart and its substructure dose. J Gannan Med Univ. (2025) 45:750–5. doi: 10.3969/j.issn.1001-5779.2025.08.004

[8] Zhang F Liu X Qiu H Li W Li X L Q . Dosimetric study of cardiac substructure with four different radiotherapy schedules after left breast cancer breast-conserving surgery. Modern Oncol. (2024) 32:1865–71. doi: 10.3969/j.issn.1672-4992.2024.10.018

[9] Mao Z Wu Q Huang C Wang Z . Dosimetric difference between VMAT and dMLC-IMRT after breast-conserving surgery for left-sided breast cancer. Chin Med Equip Joumal. (2023) 44:59–63. doi: 10.19745/j.1003-8868.2023184

[10] Li C Li S Xia B . Dosimetric study on non-coplanar volumetric modulated arc therapy technique in breast and clavicular radiotherapy following breast-conserving surgery for left-sided breast cancer. Chin J Med Phys. (2020) 37:691–5. doi: 10.3969/j.issn.1005-202X.2020.06.007

[11] Kita N Tomita N Takaoka T Okazaki D Niwa M Torii A . Clinical and dosimetric factors for symptomatic radiation pneumonitis after stereotactic body radiotherapy for early-stage non-small cell lung cancer. Clin Trans Radiat Oncol. (2023) 41. doi: 10.1016/j.ctro.2023.100648 37346273 PMC10279771

[12] Tan X Zhu S Zhou J Yuan Y Liu K Yu E . Risk factors of radiation pneumonia for 31 mediastinal lymphoma cases with drug-induced lung injury. J Pract Oncol. (2023) 38:154–61.

[13] Kuriakose J Sundararaj JJ Celine T Isaac B Mane M Singh A . Osimertinib-induced radiation recall pneumonitis. BMJ Supportive Palliative Care. (2024) 14:e1783–e5. 10.1136/spcare-2023-004291 37116943

[14] Honaryar MK Locquet M Allodji R Jimenez G Pinel B Lairez O . Cancer therapy-related cardiac dysfunction after radiation therapy for breast cancer: results from the BACCARAT cohort study. Cardio Oncol. (2024) 10:54. doi: 10.21203/rs.3.rs-4246726/v1 39187877 PMC11345963

[15] Banfill K Abravan A van Herk M Sun F Franks K McWilliam A . Heart dose and cardiac comorbidities influence death with a cardiac cause following hypofractionated radiotherapy for lung cancer. Front Oncol. (2022) 12. doi: 10.3389/fonc.2022.1007577 36303830 PMC9592751

[16] Mou Y Liang P Liu Q Du Z . Influence of imaging parameters on dosimetric parameters of lung in radiotherapy of thoracic esophageal cancer. J Pract Oncol. (2025) 40:347–53.

[17] Gradishar WJ Moran MS Abraham J Abramson V Aft R Agnese D . Breast cancer, version 3.2024, NCCN clinical practice guidelines in oncology. J Natl Compr Canc Netw. (2024) 22:331–57. doi: 10.6004/jnccn.2024.0035 39019058

[18] Darby SC Ewertz M McGale P Bennet AM Blom-Goldman U Brønnum D . Risk of ischemic heart disease in women after radiotherapy for breast cancer. (2013) 368:. doi: 10.1056/nejmoa1209825 23484825

[19] Rutqvist LE Lax I Fornander T Johansson H . Cardiovascular mortality in a randomized trial of adjuvant radiation therapy versus surgery alone in primary breast cancer. Int J Radiat Oncol Biol Phys. (1992) 22:887–96. doi: 10.1016/0360-3016(92)90784-f 1555981

[20] Jacobse JN Duane FK Boekel NB Schaapveld M Hauptmann M Hooning MJ . Radiation dose-response for risk of myocardial infarction in breast cancer survivors. Int J Radiat Oncol Biol Phys. (2019) 103:595–604. doi: 10.1016/j.ijrobp.2018.10.025 30385276 PMC6361769

[21] Thornley A Taylor K Goldsworthy S . Improving patient preparation and comfort in voluntary DIBH - single-centre quality improvement evaluation. Radiography (Lond). (2025) 31:103162. doi: 10.1016/j.radi.2025.103162 40946367

